# Evaluation of Nursing Students’ Communication Abilities in Clinical Courses in Hospitals

**DOI:** 10.5539/gjhs.v7n4p323

**Published:** 2015-01-25

**Authors:** Mahnaz Shafakhah, Ladan Zarshenas, Farkhondeh Sharif, Raheleh Sabet Sarvestani

**Affiliations:** 1 Department of Nursing, School of Nursing and Midwifery, Student research Committee, Shiraz University of Medical Sciences, Shiraz, Iran; 2 Department of Mental Health and Psychiatric Nursing, School of Nursing and Midwifery, Member of quality improvement in clinical education research center, Shiraz University of Medical Sciences, Shiraz, Iran; 3 Department of Mental Health and Psychiatric Nursing, School of Nursing and Midwifery, Community Based Nursing and Midwifery Research Center, Shiraz University of Medical Sciences, Shiraz, Iran; 4 Department of Nursing, School of Nursing and Midwifery, Fasa University of Medical Sciences, Fasa, Iran

**Keywords:** communication, nursing, student

## Abstract

**Background:**

Joint Commission on Accreditation of Healthcare Organizations (JCAHO) has established, improving communication as a priority for improving patient safety since 2006. Therefore, the present study aimed to evaluate nursing students’ communication abilities to recognize their strengths and weaknesses in communication skills.

**Method:**

This cross-sectional study was carried out in 2014. The study participants included all the nursing students who passed two semesters in Fatemeh School of Nursing and Midwifery in Shiraz, Iran. The students’ communication skills were assessed using a self-administered questionnaire. Then, the data were entered into the SPSS statistical software (v. 16) and analyzed using both descriptive (mean and percentage) and inferential statistics (Pearson correlation and ANOVA).

**Results:**

Among the 200 students who completed the questionnaires, 58% were female and 42% were male with the mean age of 21.79 years (SD=2.14). The results of Pearson correlation analysis demonstrated a significant correlation between the nursing students’ clinical communication behavior scores and treatment communication ability scores (P<0.001). The findings demonstrated that most nursing students required improvement in their communication skills in both clinical communication behavior and treatment communication ability. Besides, a significant difference was observed among the students of different terms regarding clinical communication behaviors (P≤0.05), but not concerning communication abilities. Nursing students in higher semesters had better communication skills.

**Conclusions:**

The results showed that nursing students in this university had a moderate ability in clinical and treatment communication. Thus, paying attention to standard education, curriculum revision, and adding some specific theoretical lessons for improving communication skills are mandatory during the bachelor’s degree.

## 1. Introduction

Communication is a vital element in nursing in all areas of activity, including prevention, treatment, rehabilitation, education, and health promotion (Kourkouta & Papathanasiou, 2014). If nurses want to provide a qualified professional care, they have to be able to communicate well with patients, families, physicians, and other healthcare teams. Communication with patients is the most important aspect of professional nursing whose failure can lead to ineffectiveness of nurses’ and other healthcare teams’ potentials (J. E. Liu, Wong, Xue, & Xu, 2007). Clinical communication skill is defined as communicating with patients, their family members, and other healthcare teams (J. F. Xie, Ding, Wang, & Liu, 2013). Literature showed that good communication could increase patient satisfaction and trust among nurses, patients, and family members and also decrease the patients’ blood pressure, pain, and anxiety during hospitalization (Reader, Flin, & Cuthbertson, 2007). On the other hand, failure in communication could lead to stress, errors in diagnosis, decrease of patient participation in care plans and information exchange, poor outcomes, and even death (Fallowfield, Saul, & Gilligan, 2001; Greco, Spike, Powell, & Brownlea, 2002; Leonard, Graham, & Bonacum, 2005; Meyer & Arnheim, 2002). Recent studies showed that most medical errors were not really due to defect in medical technologies or negligence of healthcare providers; rather, they were absolutely related to ineffective communication between patients and physicians (Xie et al., 2013).

Despite the effect of communication skills on the quality of nursing care and patient improvement and participation in care, the results of different researches showed that nurses were not successful in communicating with patients and families (Chant, Jenkinson, Randle, & Russell, 2002; McCabe, 2004; A. R. Zamani, Shams, Farajzadegan, & Tabaeian, 2003; A. Zamani, Shams, & Moazzam, 2004). Nurses communicated with patients for a very short period of time, they talked with patients superficially, and the communication was always about physical needs and little attention was paid to psychological needs (Krautscheid, Scholarship 2008; Sabet Sarvestani, Moattari, Nikbakht, Momennasab, & Yektatalab, 2013). This problem is evident in nursing students, too. Zay et al. in a descriptive study showed that 88.1% of nursing students had poor skills in clinical, treatment, and interpersonal communication (Xie et al., 2013). Besides, Sabzevari et al. showed that nurses’ performance was almost favorable in starting the conversation and interactive skills, intermediate in following the problems and describing the current disease, and completely inappropriate in terminating the conversation (Sabzevari, Soltani Arabshahi, Shekarabi, & Koohpayehzadeh, 2006).

Communication skills used in nursing and treatment communication behaviors are the most important skills that aim to resolve some main problems of communicating (Swinny & Brady, 2010). Expressing support, providing information and feedback, giving hope to patients, and helping them cope with anxiety are some examples of treatment communication behaviors (Elizabeth, 2006).

Since communication between nurses and patients is a core principle in patient care and Joint Commission on Aaccreditation of Health care organizations (JCAHO) has established improving communication as a priority for improving patient safety since 2006 (Schroeder, 2006), recognition of nursing students’ strengths and weaknesses in this regard is a priority for planning and modification in future. Therefore, the present study aims to evaluate and analyze different communication skills in nursing students.

## 2. Method

This cross-sectional study was carried out in Fatemeh (P. B. U. H) School of Nursing and Midwifery in Shiraz, Iran in 2014. The study participants included all the nursing students who had passed two clinical semesters in this school. The study was approved by the Research and Ethics Committee of Shiraz University of Medical Sciences and after an explanation of the study objectives, written informed consent for taking part in the study were obtained from all the students. The nursing students’ communication skills were assessed using a self-administered questionnaire which consisted of three different parts. The first part of the questionnaire was related to the demographic data. The second part which involved treatment communication behaviors contained 9 items and 42 subscales. These 9 items included sharp listening, effective transfer of information, participation, establishing harmonized relationships, preparation for communication, effective improvement, process control, ending communication work, and self-evaluation. The 42 subscales were responded through a 5-point Likert scale ranging from 1 (never) to 5 (always), but the last question was self-evaluation and scored as well, approximately good, no idea, approximately week, and week. The last part of the questionnaire was related to clinical communication behaviors and included the following 8 items: communication with schoolmates in the clinical setting and university, clinical teachers, theoretical teachers, patients’ family members, doctors, and other personnel. These items were scored through a Likert scale ranging from 1 (very comfortable) to 4 (very hard), but the last question was self-evaluation and scored as very well, well, no idea, weak, and very weak. After all, the data were entered into the SPSS statistical software (v. 16) and analyzed using descriptive and inferential statistics. Besides, P<0.05 was considered as statistically significant.

### 2.1 Reliability and Validity

At first, the original Japanese version of the questionnaire (Xie et al., 2013) was translated into the target language (Persian) by a bilingual translator. Then, the psychometric characteristics of the translated version were evaluated using two methods of random probe and committee approach. In random probe, the researcher asked 20 individuals of the target population to answer the following questions: “What do you think the question is about?” and “What do you mean?” and also determine if any item of the translated form was ambiguous or not understandable. If discrepancies were found, the related items were revised. In committee approach, on the other hand, 6 experts reviewed the clarity and linguistic appropriateness of the translated version of the questionnaire and the necessary changes were applied. The face and content validity of the items were examined with the help of 6 experts in the field of nursing and psychometric. Also, the items were assessed with respect to comprehension, ambiguity, clarity, and relation to the study objectives. In addition, the reliability of the questionnaire was confirmed using Cronbach’s alpha test.

## 3. Results

In this study, 200 students in practical clinical training completed the questionnaires. The mean age of the participants was 21.79 years (SD=2.14), 58% were female, and 42% were male. Other demographic characteristics of the participants have been presented in Table 1.

**Table 1:** Characteristics of the participants

Characteristics	Number	%
Age (year)		
18-24	186	93
25-33	14	7
Gender		
Male	84	42
Female	116	58
Semester		
2	36	18
3	46	23
4	41	20.5
5	24	12
6	46	23
8	7	3.5
Standard education		
Yes	72	36
No	131	65.5
Scores	Mean	SD
Clinical communication skills	78.60	1.34
Treatment communication skills	12.19	0.23

The participants’ mean scores of clinical communication and treatment communication abilities were 78.60±1.34 and 12.19±0.23, respectively. Besides, the result of students’ self-evaluations demonstrated that 86.2% of the participants were “good” and “approximately good” in treatment communication skills and 85.2% were “good” and “very good” in clinical communication skills. Considering the clinical communication behaviors, the participants reported that they could communicate with their schoolmates (95%), teachers (88%), patients’ family members (88%), patients (93%), nurses (91%), and doctors (72%) comfortably and very comfortably.

The results of Pearson correlation analysis demonstrated a significant correlation between the nursing students’ clinical communication behavior scores and treatment communication ability scores (r=0.352, P<0.001). Also, a significant correlation was observed between the students’ educational semester and their clinical communication behavior scores (r=0.21) and treatment communication ability scores (r=0.132) (Table 2).

**Table 2:** Correlation between the clinical communication scores and treatment communication scores

Factors	Correlation and P-value	Treatment Communication skills	Clinical Communication skills
Clinical communication skills	Correlation coefficient	0.352**	1
	P-value	≤0.001	
Treatment communication skills	Correlation coefficient	1	0.352**
	P-value		≤0.001
Semester	Correlation coefficient	0.132*	0.21*
	P-value	≤0.006	≤0.006

* *Note.* Significant at α=0.05.

** Significant at α=0.001.

However, the results of one-way ANOVA showed no significant difference between the two sexes regarding clinical communication behavior scores and treatment communication ability scores. Nonetheless, a significant difference was observed among the students of different semesters concerning clinical communication behavior scores (P<0.05), but not regarding treatment communication ability scores (Table 3). The Cronbach’s alpha of the questionnaire was 0.91.

**Table 3:** Comparison of communication skills based on different variables

	Variables	F	P-value
Clinical communication skills	Age group	3.07	0.049*
Treatment communication skills		1.12	0.328
Clinical communication skills	Semester	3.166	0.009*
Treatment communication skills		0.314	0.314
Clinical communication skills	Sex	0.103	0.174
Treatment communication skills		0.347	0.952
Clinical communication skills	Standard education	1.01	0.02*
Treatment communication skills		0.725	0.503

* *Note.* Significant at α=0.05.

## 4. Discussion

Accurate communication is a principle for nursing care and some experts have referred to this skill as the heart of nursing care (Namdar, Rahmani, & Ebrahimi, 2009). Therefore, it is imperative to teach communication skills to the nursing students who take care of patients in hospitals (Xie et al., 2013). Unfortunately, the present study findings demonstrated that most nursing students required improvement in their communication skills in both clinical communication behavior and treatment communication ability. Lambrini Kourkouta et al. (2014) stated that if nurses wanted to be successful in their work, they had to study communication and interpersonal relations through special courses and internships. They also needed to learn various aspects and applications of communication in various fields of nursing (Kourkouta & Papathanasiou, 2014).

Our study revealed that nursing students in higher semesters had better communications skills. These determinations are consistent with those obtained in World Health Organization (WHO) survey which indicated that communication ability of the nurses who graduated from colleges or universities was significantly higher compared to those who received lower educational levels (WHO, 2009).

The notable finding in this study was that most nursing students had problems communicating with physicians, which is in agreement with other previous studies. In one study, among over 1,100 nurses responding to the survey, only 43% reported feeling satisfied with their relationships with physicians and 68% doubted that physicians understood nursing responsibilities (Sirota, 2007). Moreover, JCAHO reported that communication failures among professionals caused 70% of the 2,455 reported sentinel events, resulting in about 75% of the patients’ death (Joint Commission on Accreditation of Healthcare Organizations Sentinal Event Statistics, 2004). Sirota T. (2007) indicated that nurses could improve their working relationships with physicians by continuing education, gaining specialty certifications, and participating in professional organizations, clinical research, conferences, and interdisciplinary committees (Sirota, 2007). Inter-professional education is another solution for this problem. Barr et al. (2000b) summarized the four main benefits of inter-professional education as follows: motivation to collaborate, change in attitudes and perceptions, cultivated interpersonal, group, and organizational relations, and established common value and knowledge bases (Barr, Freeth, Hammick, & Association, 2000). Hence, inter-professional education program is recommended to be enhanced in countries worldwide.

Interestingly, the current study results revealed no statistically significant difference between the two sexes regarding communications abilities. However, Merchant K. (2012) showed a significant difference between the two sexes with respect to communication styles and practices (Karima, Fall 2012). Therefore, this issue is required to be further addressed in future studies. In addition, our study showed that the students who received education could communicate better with patients, their family members, and other personnel. This indicates the important of revision and addition of some communication skills courses in the nursing curriculum. To date, no communication skills courses have been considered during bachelor’s degree education and this issue has been noted in only some lessons (Namdar et al., 2009). Thus, despite its importance, most nursing students did not learn communication skills before entering the clinical settings (Xie et al., 2013). Furthermore, the results of the present study showed a significant correlation between clinical communication skills and treatment skills scores, but this point has not been considered in our curriculums. Similar results were also obtained by Zay et al. (2012). Consistent with other studies, the current study researchers proposed that nursing communication skills could be improved through integrated teaching(J.-E. Liu, Mok, Wong, Xue, & Xu, 2007; Xie et al., 2013). Although clinical setting affects the students’ attitudes, skills, knowledge, and abilities to confront problems, it is not enough for them to learn accurate communications skills, and integrated teaching is mandatory (Henderson, Happell, & Martin, 2007; J. Xie, Ding, Wang, & Liu, 2012). Although this study was conducted on a large sample size, it relied on self-report measures, which was one of the limitations of the research. In addition, since this study was conducted on clinical nursing students in Shiraz, other universities are recommended to be evaluated and compared, as well. Moreover, given that communication ability is an important factor in health care teams, similar studies are suggested to be conducted on other healthcare personnel in hospitals.

## 5. Conclusions

Our study highlighted the importance of improving the students’ treatment and clinical communication skills. The study also showed the necessity for curriculum revision and adding some specific theoretical lessons for improving communication skills during bachelor’s degree education.
